# Riparian Meadow Response to Modern Conservation Grazing Management

**DOI:** 10.1007/s00267-017-0897-1

**Published:** 2017-06-02

**Authors:** Kristin M. Oles, Dave A. Weixelman, David F. Lile, Kenneth W. Tate, Laura K. Snell, Leslie M. Roche

**Affiliations:** 1 Department of Plant Sciences, University of California, One Shields Avenue, Davis, California 95616 USA; 2 USDA Forest Service Pacific Southwest Region, Nevada City, CA 95959 USA; 3 University of California Cooperative Extension, Susanville, CA 96130 USA; 4 University of California Cooperative Extension, Alturas, CA 96101 USA

**Keywords:** Riparian meadows, Public lands, Livestock grazing, Plant community monitoring, Long-term, United States Forest Service, Climate change

## Abstract

Riparian meadows occupy a small proportion of the public lands in the western United States but they provide numerous ecosystem services, including the production of high-quality forage for livestock grazing. Modern conservation management strategies (e.g., reductions in livestock stocking rates and adoption of new riparian grazing standards) have been implemented to better balance riparian conservation and livestock production objectives on publicly managed lands. We examined potential relationships between long-term changes in plant community, livestock grazing pressure and environmental conditions at two spatial scales in meadows grazed under conservation management strategies. Changes in plant community were not associated with either livestock stocking rate or precipitation at the grazing allotment (i.e., administrative) scale. Alternatively, both grazing pressure and precipitation had significant, albeit modest, associations with changes in plant community at the meadow (i.e., ecological site) scale. These results suggest that reductions in stocking rate have improved the balance between riparian conservation and livestock production goals. However, associations between elevation, site wetness, precipitation, and changes in plant community suggest that changing climate conditions (e.g., reduced snowpack and changes in timing of snowmelt) could trigger shifts in plant communities, potentially impacting both conservation and agricultural services (e.g., livestock and forage production). Therefore, adaptive, site-specific management strategies are required to meet grazing pressure limits and safeguard ecosystem services within individual meadows, especially under more variable climate conditions.

## 1 Introduction

Riparian meadows provide a suite of ecosystem benefits around the world. These diverse ecosystems deliver clean water, flood attenuation, nutrient sequestration, and wildlife habitat (Acreman and Holden 2013; Hatfield and LeBuhn 2007; Norton et al. 2011; Roche et al. 2012). These systems also provide essential forage for grazing livestock to produce protein for a growing human population. Society has strong contemporary expectations for stewardship of grasslands and meadows to balance agricultural goals with social, cultural, and conservation goals in a changing environment (Briske 2011). There is clear evidence that livestock can be managed to conserve and enhance ecosystem services in grazed landscapes (e.g., Davy et al. 2015; Matejkova et al. 2003; Middleton et al. 2006; Pyke and Marty 2005; Rosenthal et al. 2012). There is also clear scientific evidence that unmanaged, excessive grazing can degrade ecosystems and associated goods and services (Belsky et al. 1999; Eldridge and Greene 1994; Fleischner 1994). In a comprehensive review of the literature, Briske (2011) determined that stocking rate (i.e., livestock grazing pressure) and spatial and temporal distribution of livestock were the primary determinants of agricultural production and conservation outcomes. We argue that to balance multiple goals effectively, public lands grazing strategies must (1) establish and co-value measurable production and conservation objectives; (2) have real-time management action triggers to safeguard ecosystem services; and (3) be adaptive to accommodate spatially and temporally variable, site-specific conditions.

In the western U.S. (the West), millions of hectares of perennial grasslands, shrublands, and forestlands are held in the public domain and managed by state and federal agencies for multiple land uses, including livestock grazing. Riparian meadows account for less than 5% of the area within these landscapes, but provide disproportionate and unique ecosystem services (Bales et al. 2011; Hammersmark et al. 2010; Kuhn et al. 2011). Livestock grazing began on these landscapes during the last half of the 19th century, with numbers steadily rising to an unsustainable peak during World War I. The unregulated grazing pressure of this era led to substantial environmental degradation and impairment of ecosystem services (Sampson and Weyl 1918). In response, in the mid-1920s to early 1950s, livestock numbers were steadily reduced, grazing management areas were established and allotted to specific grazers (known as grazing allotments), and managed grazing systems were implemented to “rehabilitate” upland forage production capacities (Hormay and Evanko 1958). While these changes improved upland health and productivity (Ratliff et al. 1972), grazing plans during the rangeland rehabilitation era (mid-1950s through 1980s) did not co-value riparian areas; in fact, riparian meadows were considered “sacrifice areas” that would necessarily receive heavy livestock grazing to optimize forage harvest across grazing allotments (Meehan and Platts 1978). Thus, the interdependent plant-soil-hydrologic function of riparian meadows continued to degrade during this rangeland rehabilitation era of public lands grazing (Odion et al. 1988). At the root of this degradation was excessive defoliation of riparian meadow vegetation that reduced plant vigor, reproductive capacity, and competitiveness—shifting riparian meadow plant communities from wetland to upland or non-native species. Combined with direct impacts from livestock, these functional plant community changes reduced rooting mass and soil stability, increased soil erosion, impaired hydrologic function, and resulted in riparian desiccation; thus, diminishing a suite of regulating and supporting ecosystem services (Dwire et al. 2004; Kauffman and Krueger 1984; Kleinfelder et al. 1992; Manning et al. 1989; Micheli and Kirchner 2002; Norton et al. 2011).

**Fig. 1:**
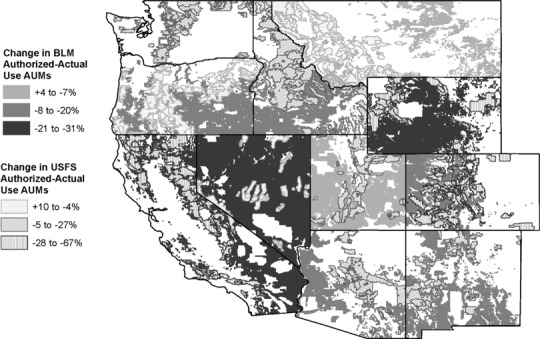
Change in animal unit months (AUM) on public lands in eleven states in the western U.S. between 2000 and 2015. The *lightest color* represents slight positive to slight negative changes in AUMs. *Darker colors* represent increasingly negative changes in AUMs. *Solid polygons* represent lands administered by the Bureau of Land Management (BLM). *Hatched polygons* represent lands administered by the U.S. Forest Service (USFS). Data were sourced from BLM and USFS annual reports (Bureau of Land Management 2016; U.S Forest Service 2016)

During the 1990s, there was a fundamental policy and on-the-ground management paradigm shift in which riparian meadows emerged as critical conservation areas—where production and conservation objectives were co-valued—rather than being relegated to “sacrifice areas” (e.g., Armour et al. 1991; U.S. Government Accounting Office 1988). This shift initiated the modern grazing era that integrated riparian meadow conservation policies and management on US federal public lands (~2000 to present). To meet conservation objectives in this modern era, livestock grazing pressure on federal grazing lands has been reduced by 15% on average across the 11 western states, with extremes of 67% and 36% in Wyoming and California, respectively (Fig. 1). A majority of these reductions were in response to implementation of annual riparian grazing utilization standards. These grazing standards are real-time management action triggers implemented annually to safeguard riparian meadows from excessive livestock utilization and to meet meadow conservation objectives (Freitas et al. 2014). Site-specific adaptive implementation of a suite of practices (e.g., herding, fencing, livestock drinking water developments) can be used to respond to action triggers, meet riparian grazing standards, and balance livestock production and multiple conservation outcomes (George et al. 2011). Modern meadow conservation objectives include maintaining and enhancing diverse, native plant communities resilient to invasion, as well as maintaining and enhancing native perennials and wetland obligate species essential to hydrologic stability.

In the late 1990s, concurrent with the initiation of the modern grazing era, the US Forest Service (USFS) in California initiated a state-wide, long-term meadow vegetation monitoring program (1) to document baseline meadow vegetation prior to the widespread implementation of these new riparian grazing standards; and (2) to examine trends in meadow vegetation following implementation of these riparian grazing standards. Our objective was to utilize data from this state-wide monitoring effort to examine whether long-term vegetation changes were significantly related to livestock grazing pressure and environmental conditions at two scales: meadow scale (i.e., ecological sites measured in hectares) and allotment scale (i.e., administration units measured in square kilometers).

## 2 Methods

### 2.1 Study Area

**Fig. 2:**
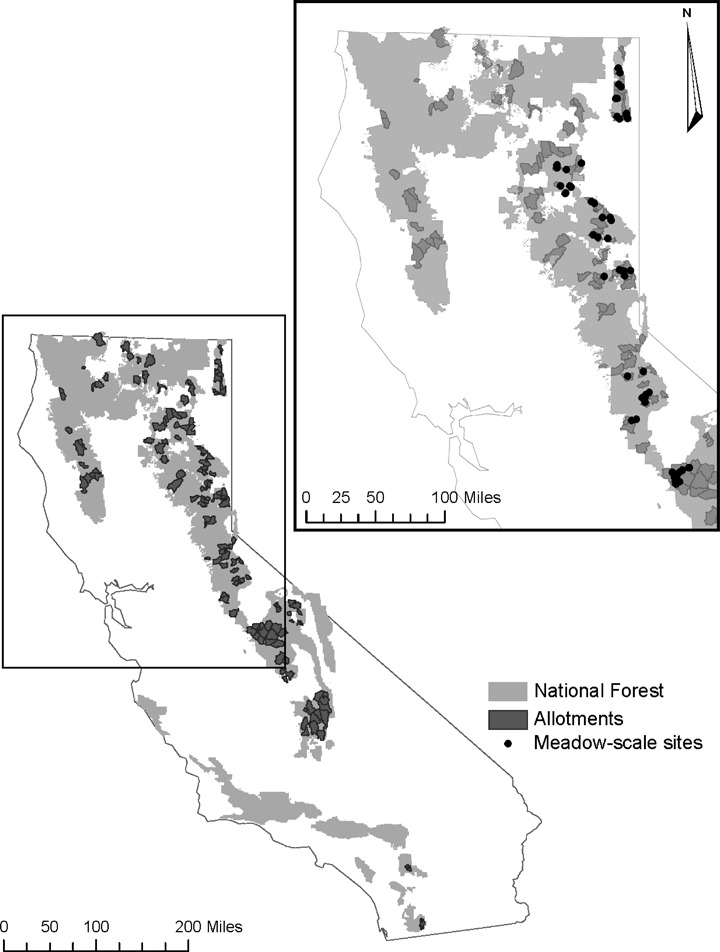
Location of allotment and meadow study areas. *Light gray* shaded areas represent National Forest lands in California. *Dark gray* shaded areas represent the allotments in the allotment scale analysis of changes in plant community and each allotment contains at least one long-term plant community monitoring site (*n* = 279). The *inset* shows a subset of monitoring sites that were selected for meadow scale analysis of changes plant community (*n* = 52)

This cross-sectional, longitudinal study was conducted across 138 grazing allotments on 16 USFS managed national forests (Fig. 2) to examine relationships between meadow scale plant community changes and allotment scale environmental conditions and livestock grazing pressure. Each allotment contains at least one long-term (i.e., more than 8 years) meadow plant community monitoring site, with a total 279 sites across the 138 allotments. All plant community monitoring sites were located within riparian meadows fed by a mix of defined stream channels, diffuse overland flow, and emergence of shallow groundwater. The 138 allotments range in elevation from 972 to 3257 m and in area from 12 to 311 km^2^ (Table 1). The majority of precipitation occurs in winter as snow.

**Table 1:** Summary of topographic, livestock grazing, and precipitation characteristics for sites at the allotment scale

	Elevation (m)	Latitude (dec. deg.)	Allotment area (km^2^)	Stocking rate–10-year average (AUM/km^2^)^a^	10-Year Cumulative (AUM/km^2^)^b^	Total annual precipitation (30-year normal, mm)
Minimum	972	32.70	12	0.0	0	312
Median	1993	38.63	99	5.8	63	787
Mean	2027	38.63	121	7.9	83	906
Maximum	3257	42.06	311	35.8	365	2091

*n* = *279*

*AUM* animal unit month

^a^ Average stocking rate during the 10 years preceding the final reading for each plant community monitoring plot, representing the average stocking rate during the plant community monitoring period.

^b^ Cumulative AUM during the plant community monitoring period standardized by allotment area.

From the 138 allotments, a subset of 34 representative grazed allotments (six national forests) were selected to examine relationships between meadow scale plant community changes and meadow scale environmental conditions and livestock grazing pressure (subset of 52 sites – Fig. 2). The subset sites were chosen as a stratified sample from the allotment scale sites to ensure representation of highly variable meadow types. This subset of 52 sites ranges in elevation from 1372 to 2490 m (Table 2). All 138 allotments are located in mountainous watersheds composed of xeric to mesic forests, with meadows and riparian corridors covering <5% of the landscape (US Forest Service 2016). Meadow plant communities are dominated by perennial grasses and grass-like species. Common species include *Carex nebrascensis* Dewey, *Juncus arcticus* Willd. subsp. *littoralis* (Engelm.) Hultén, and *Symphyotrichum spathulatum* (Lindl.) G.L. Nesom var. *spathulatum*.

**Table 2:** Summary of topographic, livestock grazing, and precipitation characteristics for sites at the meadow scale

	Elevation (m)	Latitude (dec. deg.)	Allotment area (km^2^)	Stocking Rate–10-year average (AUM/km^2^)^a^	10-year cumulative (AUM/km^2^)^b^	Total annual precipitation (30-year normal, mm)
Minimum	1373	37.33	6	1.9	19	379
Median	1961	39.90	78	8.1	86	874
Mean	1943	39.49	83	11.6	117	948
Maximum	2490	41.67	181	33.7	337	1583

*n* = *52*

*AUM* Animal unit month

^a^ Average stocking rate during the 10 years preceding the final reading for each plant community monitoring plot, representing the average stocking rate during the plant community monitoring period

^b^ Cumulative AUM during the plant community monitoring period standardized by allotment area

### 2.2 Grazing Management and Riparian Grazing Standards

During the study period (1997–2015), 100 of the 138 study allotments were grazed by beef cattle, 22 by sheep, and 16 allotments received little to no livestock grazing pressure (average annual allotment stocking rates between 0 and 5 animal unit months/km^2^). An animal unit month (AUM) is the dry weight mass of forage required to sustain one 450 kg cow for a 30-day period (360 kg). Grazing seasons and stocking rates in USFS managed grazing allotments vary depend upon elevation and local climate. Most grazing occurred from May/June through August/September, but some grazing seasons began as early as February and ended as late as December. Across all 138 allotments used for allotment scale analysis, average annual allotment stocking rates ranged from 0 to 36 AUM/km^2^ (Table 1). Stocking rates for the subset of 34 allotments used for meadow scale analysis were comparable to the full study set (Tables 1 and 2).

All grazing occurred under permits administered by the USFS, with riparian grazing standards established for meadow conservation. Specifically, three or more of the following riparian grazing standards have been in place on each grazed allotment since the initiation of plant community monitoring in 1997: (1) limits on herbaceous vegetation biomass consumption (e.g.,<40% of annual production); (2) minimum allowable residual herbaceous vegetation height (e.g.,>10 cm); (3) limits on browsing of recruiting riparian woody species (e.g.,<20% of annual leader growth); and (4) limits on livestock hoof damage to soil and streambanks (e.g.,<10% soil shearing by hooves) (Clary 1999; Clary and Leininger 2000; Clary and Webster 1990; Hall and Bryant 1995). The specific grazing management practices used in each allotment varied depending on site characteristics, but the riparian grazing standards were used as action triggers for grazing management at all study sites (US Forest Service 2004).

### 2.3 Data Collection

#### 2.3.1 Plant community metrics

Herbaceous plant community monitoring sites were established between 1997 and 2005 at 279 riparian meadows. At each site, three parallel transects, 20 m long and 5 m apart, were established and permanently marked. Twenty 0.01 m^2^ quadrats were positioned at 1 m intervals along each transect (60 total quadrats per site) during monitoring. Plant community composition data were collected by rooted species frequency methodology (Bonham 1989) at all sites approximately every 5 years between 1997 and 2013. At time of analysis, study monitoring plots had 2–7 readings (median = 3 readings), spanning 8 and 16 years of data collection (median = 10 years).

Rooted frequency data were used to calculate number of plant species (richness; S), Shannon Diversity Index (H′); and relative frequencies of forbs, non-native species, wetland obligate species, and upland species for each site and reading. S and H′ were calculated using the vegan package (Oksanen et al. 2016) in the R statistical program (R Core Team 2015). Changes over time in plant community metrics were calculated as the difference between the first and last (i.e., most recent) readings for each monitoring plot (henceforth indicated as ∆). The first and last readings vary between plots depending on when they were established and how often they are monitored.

#### 2.3.2 Livestock grazing pressure–allotment scale

Livestock grazing pressure was calculated at the allotment scale as the cumulative annual stocking pressure (AUMs/km^2^) during the plant community monitoring period (10 years preceding the most recent reading) of each site. The year of the most recent reading (i.e., year 10) ranged from 2007 to 2013. An animal unit month (AUM) is the standard metric of grazing pressure for USFS managed grazing allotments. The cumulative stocking rate (10-year cumulative AUMs/km^2^) represents the full livestock grazing pressure on the allotment over the plant community monitoring period standardized by allotment area (km^2^). Annual authorized allotment stocking rates were compiled from USFS records.

#### 2.3.3 Livestock grazing pressure–meadow scale

Because stocking rates are only assigned by the USFS at the allotment scale, meadow scale livestock grazing pressure was measured as livestock fecal density (pats/ha). Fecal density was measured at each of 52 plant community monitoring sites during the 2015 grazing season. Fecal density was measured at the end of the grazing season by counting all distinct fecal pats within three 20 m^2^ belt transects (1.3 × 15.2 m) located on the plant community monitoring plots. Because livestock fecal pats persist up to 5–10 years in these sites, fecal density represents meadow scale cumulative livestock grazing pressure (Roche et al. 2012).

#### 2.3.4 Environmental conditions–allotment scale

Relative precipitation was calculated as the mean percent of the 30-year normal precipitation (1981–2010; mm) allotments received annually (PRISM Climate Group 2015) during the monitoring period for each site within the allotment. First, both 30-year normal precipitation and total annual precipitation amounts were calculated for each allotment as the average of available values across the allotment area using gridded data from the PRISM Climate Group (PRISM Climate Group 2015). The PRISM Climate Group model uses weather station data (weighted by distance) and local geographical factors (e.g., elevation and aspect) to calculate a climate-elevation model for each 800 m grid cell (see Daly et al. 2008 for full description of PRISM methods). The 10-year mean total annual precipitation was calculated for the 10 years preceding the most recent reading for each plot within the allotment. The year of the most recent reading (i.e., year 10) ranged from 2007 to 2013. Then, using allotment 30-year normal total annual precipitation values, relative precipitation was calculated as

$${\rm{Relative}}\,{\rm{precipitation = }}\left({\frac{{10\hbox{-}{\rm{ year}}\,{\rm{mean}}\,{\rm{total}}\,{\rm{annual}}\,{\rm{precipitation}}}}{{30\hbox{-}{\rm{year}}\,{\rm{normal}}\,{\rm{total}}\,{\rm{annual}}\,{\rm{precipitation}}}}} \right) \times 100$$

Therefore, relative precipitation values below 100% indicate the 10-year period was drier than average for the respective allotments.

#### 2.3.5 Environmental conditions–meadow scale

During the 2015 grazing season, site wetness was also recorded for the subset of 52 plant community monitoring sites. Site wetness was rated on a scale of 1–5, where 1 is the driest site and 5 is the wettest site (Roche et al. 2012; Roche et al. 2014) observed in this study. Plant community, soil characteristics, and hydrologic characteristics observed during data collection were all used to determine the site wetness rank for each site. For example, a site dominated by wetland obligate species on organic soils and visible standing water at the end of the grazing season would represent a 5 rank. A relatively drier grass/forb-dominated site on mineral soils would represent a 1 rank. Meadow scale relative precipitation was calculated following the methods outlined for allotment scale (Section 2.3.4) (PRISM Climate Group 2015). Meadow scale 30-year normal precipitation and total annual precipitation amounts were queried from the PRISM Climate Group (PRISM Climate Group 2015) for a single geographic point—the coordinates of the plant community monitoring site within each meadow—using the raster package (Hijmans 2015) in the R statistical program (R Core Team 2015).

### 2.4 Statistical Analysis

#### 2.4.1 Allotment scale

Linear regression analysis was used to test for associations between changes (∆) in plant community metrics at each monitoring site, and allotment scale livestock grazing pressure and environmental conditions during the study period (279 sites across 138 grazing allotments). Backward step-wise selection was used to determine final models, with *p* < 0.1 (using a Bonferroni correction of <0.016 to account for six analyses) required for significance. The Bonferroni correction protects against Type I error when multiple comparisons are made by setting a lower threshold for significance. Allotment scale relationships with *p* < 0.016 are reported as “statistically significant”, while relationships with *p* < 0.1 are reported as “apparent”. Initial full model independent variables were: allotment elevation (m), relative precipitation, cumulative stocking rate (AUM/km^2^), and the cumulative stocking rate by relative precipitation interaction term. Individual models were analyzed for six plant community response metrics (dependent variables): ∆S; ∆H′; and ∆ relative frequency (%) of forbs, non-native species, wetland obligate species, and upland species. Regression analyses were conducted using the R statistical program (R Core Team 2015), and standard diagnostics were used to confirm that assumptions were met.

#### 2.4.2 Meadow scale

Meadow scale analyses were conducted on a subset of 52 monitoring sites located across 34 grazing allotments (Fig. 2). In addition to the six meadow scale plant community change metrics described in Section 2.3.1, changes in overall meadow plant community composition during the study period were estimated via non-metric multidimensional scaling (NMDS) using a Euclidean distance matrix. Solutions were tested for 1 through 7 dimensions and a plot of final stress vs. number of dimensions was used to determine the optimal number of dimensions (McCune and Grace 2002). Composition was quantified using NMDS scores from the first dimension of the Euclidean distance matrix (the dimension that explains the most variation in the data) and changes in composition were calculated as the difference between the first and last years for each monitoring plot. NMDS analysis was completed using the metaMDS function in the vegan package (Oksanen et al. 2016) in the R statistical program (R Core Team 2015). Permutational multivariate analysis of variance (perMANOVA) with a Euclidean distance matrix was used to test for shifts in overall plant community composition between first and last years. This analysis was conducted using the adonis function in the vegan package of the R statistical program (Oksanen et al. 2016; R Core Team 2015).

Linear regression analysis was used to test for associations between changes (∆) in plant community metrics at each monitoring site, and meadow (site) scale livestock grazing pressure and environmental conditions during the study period (52 sites within 34 grazing allotments). Backward step-wise selection was used to determine final models, with *p* < 0.1 (using a Bonferroni correction of < 0.014 to account for seven analyses) required for significance. Meadow scale relationships with *p* < 0.014 are reported as statistically significant, while relationships with *p* < 0.1 are reported as apparent. Initial full model independent variables were: meadow elevation (m), relative precipitation, wetness ranking, fecal density (pats/ha), and the fecal density by relative precipitation interaction term. Individual final models were analyzed for seven plant community response metrics (dependent variables): ∆S; ∆H′; ∆ species composition (calculated from NMDS scores of first and last reading years); and ∆ relative frequency (%) of forbs, non-native species, wetland obligate species, and upland species. Regression analyses were conducted using the R statistical program (R Core Team 2015), and standard diagnostics were used to check conformity with assumptions.

## 3 Results

### 3.1 Allotment Scale

**Fig. 3:**
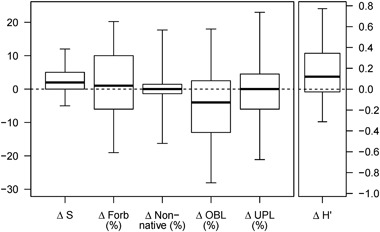
Long-term change (Δ) in allotment scale plant community metrics in 279 plant community monitoring plots in riparian meadows across California (*n* = 279). Metrics are species richness (S), diversity (H′), and the relative frequencies of forb, non-native, wetland obligate (OBL), and upland species (UPL). *Dark lines* represent the median. *Top* and *bottom box* boundaries represent the 75th and 25th percentiles, respectively. *Top* and *bottom* *whiskers* represent the 95th and 5th percentiles, respectively

At the allotment scale, average precipitation during the 10 years preceding the most recent readings ranged from 75% to 110% of 30-year normal with a mean value of 94%. The allotment-scale median total annual precipitation during the study period was slightly lower than the median total annual precipitation during the preceding decade (data not shown). Median changes for non-native species and upland species were 0 (i.e., no change between first and last year) across all 138 allotments (279 monitoring sites) (Fig. 3). Median changes were positive for S, H′, and forbs, while wetland obligate species median change was downward. Neither highly or moderately invasive species (California Invasive Plant Council 2016) were abundant with median relative frequencies and changes of 0. Moderately invasive species were observed in 22 allotments (30 of 279 sites), and highly invasive species were observed in 17 allotments (25 of 279 sites).

**Fig. 4:**
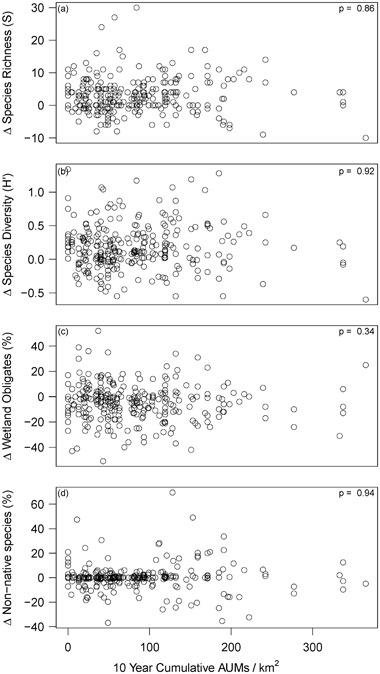
Allotment scale changes (Δ) in (a) species richness (S), (b) species diversity (H′), and the relative frequencies of (c) wetland obligate and (d) non-native species (%) by the 10-year cumulative allotment stocking rate (AUM/km^2^; *n* = 279). As indicated by the *p* values listed in each panel, no significant relationships were found between these plant community metrics and cumulative stocking rate at the allotment scale (Bonferroni-corrected *p* < 0.016)

**Fig. 5:**
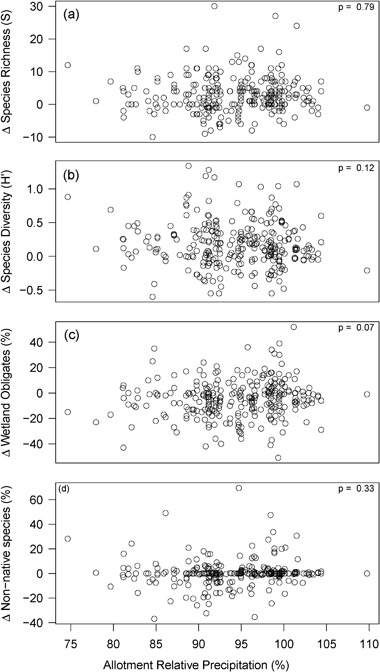
Allotment scale changes (Δ) in (a) species richness (S), (b) species diversity (H′), and the relative frequencies of (c) wetland obligate and (d) non-native species (%) by the 10-year average relative precipitation (percent of 30-year normal annual precipitation; *n* = 279). As indicated by the *p* values listed in each panel, no significant relationships were found between these plant community metrics and cumulative stocking rate at the allotment scale (Bonferroni-corrected *p* < 0.014). One apparent relationship (i.e., *p* < 0.1) was found between changes in wetland obligate species and relative precipitation

No significant (Bonferroni correction of *p* < 0.016) relationships were found between allotment scale livestock grazing pressure, relative allotment scale precipitation, or their interaction term with any of the plant community metrics examined (∆S; ∆H′; and ∆ relative frequency of forbs, non-native species, wetland obligate species, and upland species) (Figs. 4 and 5). There was an apparent, but not statistically significant, positive relationship (*p* = 0.07, slope = 0.28, adjusted *r* ^2^ = 0.008) between precipitation and changes in relative frequency of wetland obligate species (Fig. 5c). There was a significant negative relationship (*p* = 0.009, slope = −0.004, adjusted *r* ^2^ = 0.02) between elevation and changes in relative frequency of upland species; and an apparent, but not statistically significant, negative relationship (*p* = 0.07, slope = −0.003, adjusted *r* ^2^ = 0.008) between elevation and changes in relative frequency of non-native species (not shown). No other significant or apparent relationships were found.

### 3.2 Meadow Scale

**Fig. 6:**
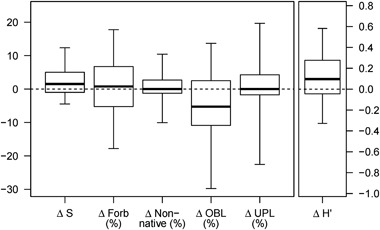
Long-term trends (Δ) in meadow scale plant community metrics in 52 grazed meadows. Richness (S), diversity (H′), and the relative frequencies of forb, non-native, wetland obligate (OBL), and upland (UPL) species. *Dark lines* represent the median. *Top* and *bottom box* boundaries represent the 75th and 25th percentiles, respectively. *Top* and *bottom* *whiskers* represent the 95th and 5th percentiles, respectively

At the meadow scale, average precipitation during the 10 years preceding the most recent reading ranged from 59% to 96% of 30-year normal with a mean value of 82%. The meadow-scale median total annual precipitation for the study period was similar to the median total annual precipitation during the decade prior to the study period (data not shown). The perMANOVA analysis indicated that plant community composition was significantly different between the first and last readings (*p* < 0.001), which indicates that plant communities in the study meadows have shifted in composition during the study period. Median changes across the subset of 52 monitoring sites for non-native species and upland species were 0 (i.e., no change between first and last year) (Fig. 6). Median changes in S, H′, and forbs were positive, while changes in wetland obligate species was negative. Neither highly or moderately invasive species (California Invasive Plant Council 2016) were abundant with median relative frequencies and changes of 0.

**Fig. 7:**
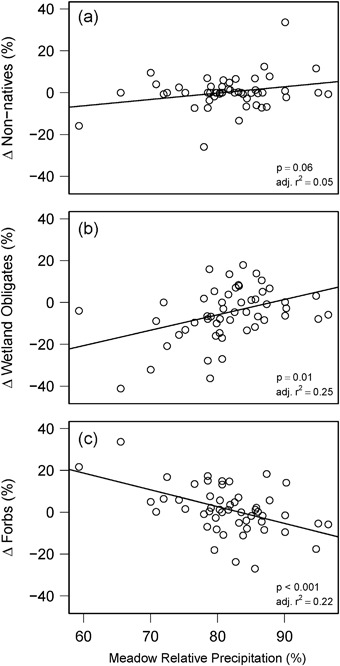
Meadow scale changes (Δ) in the relative frequencies (%) of (a) non-native species, (b) wetland obligate species, and (c) forbs by precipitation as a percent of long-term normal levels (*n* = 52). The effect of precipitation was positive for wetland obligate species and negative for forbs (Bonferroni-corrected *p* < 0.016). There was an apparent (i.e., *p* < 0.1) positive effect of precipitation for non-native species. *P* values are presented for the effect of meadow relative precipitation (10-year average annual precipitation percent of 30-year normal annual precipitation) in each model

**Fig. 8:**
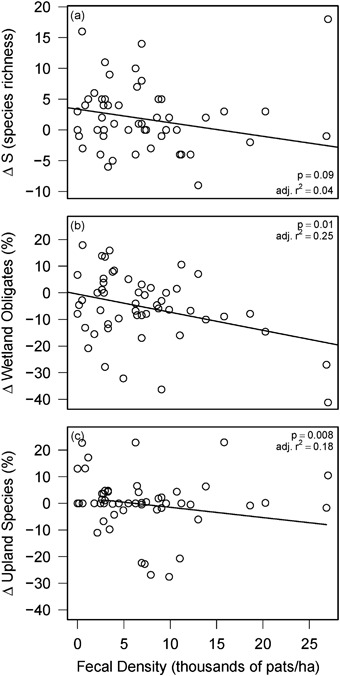
Meadow scale changes (Δ) in species richness (S) and the relative frequency (%) of wetland obligate species and upland species by fecal density (*n* = 52). Fecal density had a significant negative effect of on changes in wetland obligate species (Bonferroni-corrected *p* < 0.016). The fecal density by relative precipitation interaction was significant for changes in upland species. There was an apparent (i.e., *p* < 0.1) negative effect of fecal density on changes in species richness. In *panels* **a** and **b**, *p* values are presented for the fecal density effect. In *panel* **c**, the *p* value shown is for the interaction of fecal density and relative precipitation and the *line* represents the effect of fecal density at the median relative precipitation value (81.7% of 30-year normal annual precipitation). The *line* has been clipped to the range of fecal densities observed at sites receiving approximately 81.7% relative precipitation

Significant (Bonferroni correction of *p* < 0.014) relationships were found for three of the seven plant community metrics analyzed (Table 3). However, adjusted *r* ^2^ values (0.18 to 0.25) indicate that, while these regression models were significant, substantial variation was left unexplained by meadow scale livestock grazing pressure and environmental conditions observed during the study period. Relative precipitation was significantly related to changes in forbs (*p* < 0.001) and wetland obligate species (*p* = 0.01), as well as apparently related to changes in non-native species (*p* = 0.06) (Fig. 7, Table 3). Fecal density was significantly and negatively related to changes in wetland obligate species (*p* = 0.01) and upland species (*p* = 0.01) (Fig. 8b and c, Table 3). A significant (*p* = 0.01) fecal density by relative precipitation interaction indicated a more positive association between fecal density and change in upland species for sites experiencing drier conditions (Table 3). Fecal density was apparently (*p* = 0.09) negatively related to changes in species richness (Fig. 8a). Meadow site wetness rank (1 through 5) was apparently (*p* = 0.07) associated with changes in meadow plant community composition (calculated from NMDS scores of first and last reading years) (Table 3). Plant community composition scores (via NMDS) are reported in Online Resource 1.

**Table 3:** Meadow-scale analysis of relationships between plant community trends and management and environmental factors on USFS lands in California

Response	Parameter	Coefficient	*P*-value
ΔS (richness) (adj. *r* ^2^ = 0.04)	Elevation	*ns*	0.56
	Site wetness rank	*ns*	0.80
	Precipitation percent of normal	*ns*	0.37
	Fecal pats	1.32	0.09
	Precipitation * fecal Pats	*ns*	0.61
ΔH′ (diversity)	Elevation	*ns*	0.37
	Site wetness rank	*ns*	0.45
	Precipitation percent of normal	*ns*	0.16
	Fecal pats	*ns*	0.63
	Precipitation * fecal pats	*ns*	0.70
ΔForbs (%) (adj. *r* ^2^ = 0.22)	Elevation	*ns*	0.34
	Site wetness rank	*ns*	0.71
	Precipitation percent of normal	−5.737	< 0.001*
	Fecal pats	*ns*	0.37
	Precipitation * fecal pats	*ns*	0.10
ΔNon-native species (%) (adj. *r* ^2^ = 0.05)	Elevation	*ns*	0.37
	Site wetness rank	*ns*	0.95
	Precipitation percent of normal	2.173	0.06
	Fecal pats	*ns*	0.39
	Precipitation * fecal pats	*ns*	0.79
ΔWetland obligate species (%) (adj. *r* ^2^ = 0.25)	Elevation	*ns*	0.65
	Site wetness rank	*ns*	0.50
	Precipitation percent of normal	4.458	0.01*
	Fecal pats	−4.260	0.01*
	Precipitation * fecal pats	*ns*	0.35
ΔUpland species (%) (adj. *r* ^2^ = 0.18)	Elevation	*ns*	0.89
	Site wetness rank	*ns*	0.81
	Precipitation percent of normal	−4.604	0.32
	Fecal pats	−2.732	0.01*
	Precipitation * fecal pats	−6.2772	0.01*
ΔSpecies composition (adj. *r* ^2^ = 0.05)	Elevation	*ns*	0.39
	Site wetness rank	1.257	0.07
	Precipitation percent of normal	*ns*	0.64
	Fecal pats	*ns*	0.36
	Precipitation * fecal pats	*ns*	0.21

*n* = *52*

Δ indicates the trend in a given plant community metric during the monitoring period. Final model coefficients are centered on the mean and scaled to one standard deviation. Coefficients are given for parameters with apparent relationships with responses (*p* < 0.1). “ns” indicates no apparent relationship between the parameter and response (*p* ≥ 0.1) and the reported *p*-value is the final *p*-value for that parameter before it was dropped from the model

*indicates the parameter was significant using Bonferroni corrected *p* < 0.016

## 4 Discussion

Plant community composition across the 279 riparian meadows enrolled in this study has been changing over the past decade, with substantial variation in magnitude and direction of change found between meadows (Fig. 3). Overall, median changes in plant community metrics suggest that species diversity and richness are increasing, and that the non-native and upland species components of these meadow communities have been constant. Invasive plant species frequency has remained at low levels throughout this period, with increases in overall species richness and diversity driven by increases in native forbs. These outcomes are congruent with meadow conservation objectives. The largest observed functional change was a reduction in the relative frequency of wetland obligate species during the study period. The majority of the meadows studied received precipitation below the 30-year normal throughout the study decade.

Associations between livestock grazing pressure and changes in plant community varied across spatial scales. Our results suggest that allotment scale livestock grazing pressure (i.e., stocking rate) is currently at a level that balances production and conservation goals at the allotment scale. During the study period, total grazing pressure declined 36% across USFS administered grazing allotments in California, second only to Wyoming in the 11 western states (Fig. 1). The reduced allotment scale grazing pressure observed in this study was not associated with changes in plant community metrics (Fig. 4). However, there were slight negative associations between meadow scale grazing pressure and changes in three of the seven metrics examined (Fig. 8, Table 3). This suggests that at the current reduced stocking rates, heterogeneity of livestock use within an allotment is a significant driver of changes in meadow plant community. Meadow scale results confirm the importance of adaptively managing annual grazing pressure on individual meadows within allotments (Clary 1999; Freitas et al. 2014; George et al. 2011) even when allotment stocking rates are relatively low. Grazing management and distribution strategies (e.g., off stream water development) can be effective in limiting cattle use of sensitive habitats like riparian areas (Johnson et al. 2016). Implementation and monitoring of livestock grazing distribution strategies to meet riparian grazing standards in all meadows within allotments is key to obtaining conservation objectives.

Meadow scale monitoring is especially important to adjust grazing management as climate conditions change over time. Precipitation timing and frequency is expected to become more unpredictable, especially at higher elevations (Cayan et al. 2008; Maurer 2007). Most studies predict that these precipitation changes will lead to earlier spring runoff, reduced annual snowpack and steam flow, and more winter rainfall-runoff—resulting in a longer and drier summer growing season (Miller et al. 2003; Null et al. 2010; Mote et al. 2005; Stewart et al. 2005). These changes would likely result in reduced extent and duration of soil saturation and anaerobic conditions in wetter meadows. Results from this study (Table 3) and previous studies (Allen-Diaz 1991; Dwire et al. 2004; Freitas et al. 2014) show that plant community composition in these meadows is strongly linked to hydrological processes (e.g., site wetness and precipitation). Shifts in plant community composition associated with hydrological processes are important to consider in grazing management decisions as climate patterns become more variable, even for plant community metrics that were not associated with grazing pressure (e.g., forbs and non-native species, Table 3). Changes in precipitation amount and timing, as well as any associated effects on stream flow and water table dynamics, will likely lead to changes in plant community structure and, consequently, the timing, amount, and quality of forage available for livestock grazing (Izaurralde et al. 2011; Null et al. 2010). Additionally, grazing pressure interacted with precipitation for a plant community metric at the meadow scale (Δ upland species, Table 3), suggesting that plant community responses to grazing could also become more varied as climate changes. Because individual meadows are likely to experience and respond to changing climate conditions in site-specific ways, site-level monitoring and grazing management will be important to assess meadow trends and response to grazing.

Since the mid-20th century, public grazing lands policy and management paradigms have moved to increasingly integrate agricultural and conservation goals. In the modern public lands grazing era, riparian meadows are part of a working landscape that is managed to balance a multitude of economic, social, and ecological goals. Our work, and the work of others (Clary and Leininger 2000; Clary and Webster 1990; Freitas et al. 2014; Hall and Bryant 1995), suggests that current riparian grazing utilization limits can effectively protect these meadow resources. Adaptive implementation of site-specific riparian grazing conservation strategies to meet these limits is critical to safeguard ecosystem services, particularly under increasingly variable and changing environmental conditions.

## Electronic supplementary material

Supplementary Information
