# Supplementary figures and images for: Riparian Meadow Response to Modern Conservation Grazing Management
Source: Environ Manage. 2017 Jun 2;60(3):383–95. doi: 10.1007/s00267-017-0897-1 (PMC5544782; doi:10.1007/s00267-017-0897-1)

NMDS2

20  
10  
0  
-10  
-20

-40

-20

NMDS1

0

20

40

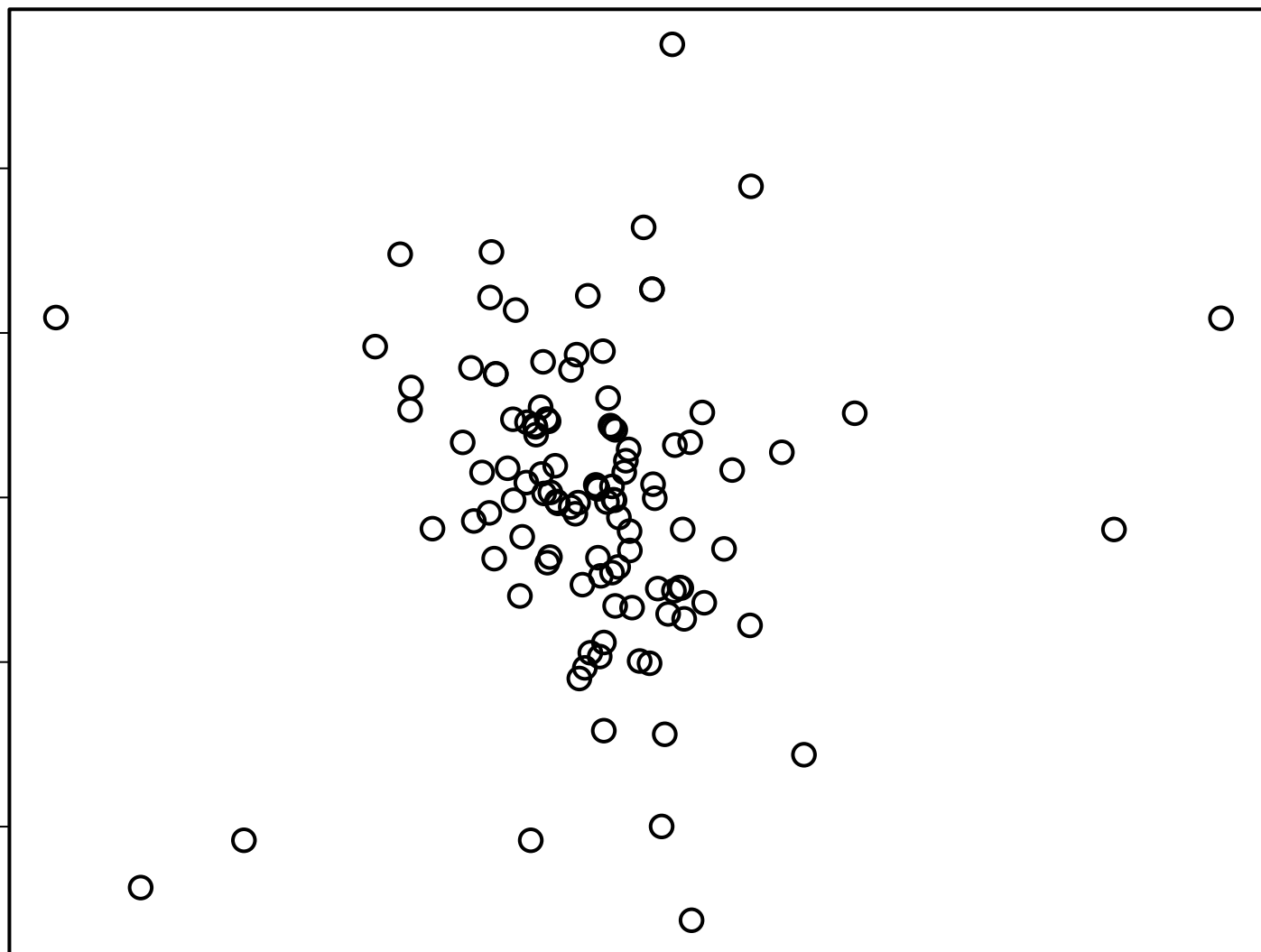

Supplement: Supplementary file 1 — Supplementary Information [file 267_2017_897_MOESM1_ESM.pdf]
